# Evaluation of Clinical Predictors for Major Outcomes in Patients Hospitalized With COVID-19: The Potential Role of the Rothman Index

**DOI:** 10.7759/cureus.28769

**Published:** 2022-09-04

**Authors:** Diego Moguillansky, Omar M Sharaf, Phoebe Jin, Ronny Samra, Jaimie Bryan, Natalia I Moguillansky, Jorge Lascano, Juan N Kattan

**Affiliations:** 1 Department of Pediatrics, University of Florida Health Congenital Heart Center, Gainesville, USA; 2 College of Medicine, University of Florida, Gainesville, USA; 3 Department of Medicine, Division of Pulmonary Critical Care and Sleep Medicine, University of Florida Health, Gainesville, USA; 4 Department of Medicine, Division of Hospital Medicine, University of Florida Health, Gainesville, USA

**Keywords:** predictive models of mortality, risk of covid 19 mortality, covid-19 pandemic, mechanical ventilator, medical icu, risk assessment tools, sars-cov-2, rothman index, covid-19, coronavirus disease

## Abstract

Introduction

The Rothman Index (RI, PeraHealth, Inc. Charlotte, NC, USA) is a predictive model intended to provide continuous monitoring of a patient's clinical status. There is limited data to support its use in the risk stratification of patients infected with severe acute respiratory syndrome coronavirus 2 (SARS-CoV-2). We hypothesized that low admission RI scores would correlate with higher rates of adverse outcomes in patients hospitalized for coronavirus disease 2019 (COVID-19).

Methods

Medical records of adult patients admitted to a single 1,200-bed tertiary academic center were retrospectively reviewed for demographic data, baseline characteristics, RI scores, admission to intensive care unit (ICU), need for mechanical ventilation, and inpatient mortality. Statistical analyses were performed using STATA statistical software, version 17 (Stata Corp LLC, College Station, TX, USA). Continuous variables were analyzed using the Mann-Whitney test, and categorical variables were analyzed using Fisher’s exact test. Both univariate and multivariate analyses were performed. A p-value <0.05 was considered statistically significant.

Results

Median admission RI score for the entire cohort was 63.0 (IQR 45.0 - 77.1). The cohort was divided by admission RI into a low-risk group (RI ≥70; n=70) and a high-risk group (RI <70; n=107). Compared to patients with low-risk RI, patients with high-risk RI had higher mortality (95.2%, 95% CI: 85.8 - 105 vs 4.8%, 95% CI: -5 - 14.2, p < 0.01), were more likely to require ICU admission (90.2%, 95% CI: 81.9 - 98.5 vs 9.8%, 95% CI: 1.5 - 18.1, p < 0.01) and mechanical ventilation (89.7%, 95% CI: 78.3 - 101 vs 10.3%, 95% CI: -1 - 21.7, p < 0.01), and had a longer median hospital length of stay (12 days, 95% CI: 9 - 14 vs 5 days, 95% CI: 4 - 7, p < 0.01).

Conclusions

High-risk RI was associated with increased admission to the ICU, mechanical ventilation, and mortality. These results suggest that it may be used as a tool to aid provider judgment in the setting of COVID-19.

## Introduction

Since late 2019, severe acute respiratory syndrome coronavirus 2 (SARS-CoV-2) has spread to all corners of the world causing a wide range of clinical diseases ranging from asymptomatic infections to severe coronavirus disease 2019 (COVID-19), including acute respiratory distress syndrome (ARDS). Estimates for patients who develop severe symptoms requiring admission to the intensive care unit (ICU) range from 5% to 20% [1] and around 1.56% of patients ultimately succumb to the disease [2].

Several factors including male gender, age, and presence of comorbidities (diabetes, hypertension, liver and heart disease) have been linked to increased mortality from the virus [3]. However, conflicting reports exist from different regions, assigning different weights to these predictors of outcome [4]. While some or all of these variables may be associated with adverse outcomes, they do not reflect the acuity of the presentation.

The Rothman Index (RI, PeraHealth, Inc. Charlotte, NC, USA) is a predictive model which uses continuous measurements of patient data from 26 non-static variables to measure physiologic acuity. A score based on vital signs, nursing assessments, laboratory results, and cardiac rhythm is calculated by measuring deviation from standard values, with a maximum score of 100 which signifies no deviation from standard values. A decrease in score correlates to a decrease in patient health. The RI was designed to be a score that is applicable to all types of patients, regardless of diagnosis, procedure, or environment [5]. The intention of the RI is to give providers quantifiable, continuous monitoring of a patient’s clinical status that is automatically generated by the electronic medical record (EMR).

Previously, studies have shown that RI was useful in early detection of deterioration of clinical status, resulting in reduced mortality and discharge to hospice [6]. It has shown predictive value in unplanned readmissions [7], post-discharge adverse events [5], 24-hr mortality, and one-year mortality [8,9]. However, other studies have not shown RI to effectively predict deterioration inwards [10] and it was not shown to be superior to a physician's judgment [11]. In other investigations, RI performs better than strictly vital sign-based acuity scores, such as the Modified Early Warning Score (MEWS), in identifying hospitalized patients who are at risk of dying within 24 hours [12].

A recent study conducted by PeraHealth, the developers of RI, has shown that RI has some use in risk stratification for patients with COVID-19; however, the accuracy and effectiveness of its implementation are of unknown significance [13].

Unique to the RI is its incorporation of nursing assessment into the overall score. The frequent head-to-toe monitoring of the patient can account for functional deteriorations that precede changes in vital signs or lab values, potentially making it more sensitive than other acuity scores [8,12]. This tool could allow for earlier detection of decline and earlier intervention, making it particularly useful in the setting of a novel disease when specific indicators of a worsening disease state are still unknown and physician judgment is still building. This study aims to assess the association of the RI in patients admitted to the hospital for COVID-19 with three clinically important outcomes: the need for mechanical ventilation, ICU admission, and inpatient mortality.

## Materials and methods

All consecutive adult patients admitted to the hospital who tested positive for SARS-CoV-2 from April 2020 to July 2020 were reviewed in this retrospective cohort study at a single 1,200-bed tertiary academic center in North-central Florida. Waiver for written informed consent was obtained from the University of Florida Health System Institutional Review Board. Compliance with the World Medical Association Declaration of Helsinki was maintained during this retrospective cohort study.

Infection with SARS-CoV-2 was confirmed through nucleic acid amplification testing of nasopharynx swabs using real-time reverse-transcriptase polymerase chain reaction assays, carried out according to validated protocols [14].

EMRs were reviewed to collect data on demographics, baseline characteristics, admission RI scores, and outcomes of interest; the primary outcome was inpatient mortality, and secondary outcomes were the need for mechanical ventilation and admission to the ICU. High-risk RI was defined as <70 based on prior literature [6]. In order to dichotomize the groups for statistical analysis, RI ≥70 was considered low-risk. Patients who were admitted for a primary problem unrelated to COVID-19 with incidentally positive SARS-CoV-2 tests were excluded from the analysis. Incidentally positive was defined as having a positive SARS-CoV-2 nucleic acid amplification test ordered per hospital protocol but not exhibiting any of the most common symptoms of COVID-19, such as fever, cough, or dyspnea [15] and having a primary admission diagnosis that was not COVID-19.

As our data was not normally distributed, we utilized non-parametric statistics. Continuous variables were analyzed using the Mann-Whitney test and were presented using medians and interquartile ranges. Categorical variables were analyzed using Fisher’s exact test and were presented as percentages with 95% CI. Variables significant at the 0.10 level on univariate analysis were selected for multivariate analysis. Multivariate analysis was performed with a non-parametric logistic regression with odds ratios and CIs. Continuous variables such as age were recoded into quintiles for ease of analysis. Multivariate analysis results were presented as odds ratios with 95% CI and two-sided p-values. Alternative logistic regression models including only variables significant at the 0.05 level on univariate analysis were also performed to assess for robustness and since they yielded the same final results, they were not included in this paper. Two-sided p-values < 0.05 were considered statistically significant in multivariate analysis. No imputations were made for missing data. Statistical analyses were performed using STATA statistical software, version 17 (Stata Corp LLC, College Station, TX, USA) [16].

## Results

A total of 210 patients were admitted to the hospital with a positive SARS-CoV-2 test during the time frame of the study. Thirty-one patients incidentally tested positive during admission for another primary diagnosis. These 31 patients did not exhibit the most common symptoms of COVID-19 [15] and hence were excluded from the analysis. Of the 179 patients admitted for COVID-19, RI could not be calculated in two patients.

Demographic data and baseline characteristics are shown in Table 1. The median age for the entire cohort was 58.4 years (IQR 45.1 - 70.2) and 39% of the cohort was male. No statistically significant differences were found for age or gender when compared for the primary (inpatient mortality) or secondary outcomes (need for mechanical ventilation, admission to the ICU).

**Table 1:** Univariate Analysis of Predictors of Intensive Care Unit Admission, Mechanical Ventilation, and In-Hospital Mortality in Patients Admitted with COVID-19 Values displayed as percentages with 95% confidence intervals. ICU, intensive care unit; IQR, interquartile range.

Variable	ICU Admission (n=51)	No ICU Admission (n=128)	p-value	Mechanically Ventilated (n=29)	Not Mechanically Ventilation (n=150)	p-value	Deceased (n=21)	Survived (n=156)	p-value
High Risk Rothman index	90.2 (81.9 - 98.5)	48.4 (39.6 - 57.2)	0.000	89.7 (78.3 - 101.0)	54.7 (46.6 - 62.8)	0.000	95.2 (85.8 - 105)	55.8 (47.9 - 63.6)	0.000
Age years (Median, IQR)	60.7 (43.5 - 67.3)	58 (45.6 - 71.65)	0.062	62.7 (43.5 - 67.3)	57.9 (45.6 - 70.4)	0.049	57.8 (45.1 - 67.5)	67.4 (58.3 - 78.2)	0.094
Elevated Troponin I	32.0 (18.8 - 45.2)	23.1 (15.1 - 31.2)	0.248	34.5 (16.7 - 52.2)	24.0 (16.6 - 31.5)	0.250	42.9 (21.0 - 64.7)	23.4 (16.2 - 30.5)	0.066
Renal Replacement Therapy	13.7 (4.1 - 23.3)	5.5 (1.49 - 9.45)	0.118	20.7 (5.6 - 35.8)	5.33 (1.7 - 9.0)	0.013	6.96 (2.95 -11.0)	14.3 (-1.16 - 29.7)	0.216
Obesity	62.7 (49.3 - 76.2)	48.4 (39.7 - 57.2)	0.098	62.0 (44.0 - 80.2)	50.7 (42.6 - 58.8)	0.312	57.1 (35.3 - 78.9)	51.9 (44.0 - 59.8)	0.817
CAD/CHF	27.5 (15.0 - 39.9)	28.9 (21.0 - 36.8)	1.000	34.5 (16.8 - 52.2)	27.3 (20.1 - 34.5)	0.501	38.1 (16.7 - 59.5)	27.2 (20.2 - 34.2)	0.311
Hyperlipidemia	43.1 (29.3 - 57.0)	36.7 (28.3 - 45.2)	0.497	44.8 (26.3 - 63.4)	37.3 (29.5 - 45.2)	0.533	47.6 (25.6 - 69.7)	37.3 (29.7 - 45.0)	0.475
Hypertension	66.7 (53.5 - 79.8)	61.7 (53.2 - 70.2)	0.608	69.0 (51.7 - 86.2)	62.0 (54.2 - 69.9)	0.534	71.4 (51.5 - 91.4)	62.0 (54.4 - 69.7)	0.477
Diabetes Mellitus	42.0 (28.1 - 55.9)	43.8 (35.1 - 52.4)	0.868	50.0 (31.0 - 69.0)	42.0 (34.0 - 50.0)	0.534	50.0 (27.4 - 72.6)	42.4 (34.6 - 50.2)	0.633
Reduced RV Systolic Function	21.2 (67.4 - 35.7)	83.3 (-3.2 - 19.9)	0.277	21.7 (4.1 - 39.3)	11.7 (0.5 - 23.0)	0.461	21.4 (-1.4 - 44.2)	14.0 (3.2 - 24.7)	0.674
Reduced LV Systolic Function	21.2 (6.7 - 35.7)	25.0 (6.9 - 43.1)	0.759	17.4 (12.0 - 33.6)	26.5 (11.0 - 41.8)	0.529	28.6 (3.5 - 53.6)	20.9 (8.4 - 33.5)	0.715
Male	39.2 (25.6 - 52.8)	39.1 (30.5 - 47.6)	1.000	44.8 (26.3 - 63.4)	38.0 (30.2 - 45.9)	0.536	42.9 (21.0 - 64.7)	38.6 (31.0 - 46.3)	0.813
Liver Disease	3.9 (-1.5 - 9.4)	10.2 (4.9 - 15.5)	0.238	69.0 (-2.6 - 16.4)	8.7 (4.2 - 13.2)	1.000	4.8 (-4.6 - 14.2)	8.9 (4.4 - 13.3)	1.000

Age achieved a p-value of < 0.10 for all three outcomes and hence was included in all multivariate analyses. Obesity (defined as body mass index ≥30) was included in the multivariate analysis for ICU admission after achieving a p-value of <0.10 in univariate analysis. It was not included in the models for mortality or mechanical ventilation as it did not achieve a p-value of <0.10 in univariate analysis for these variables. Statistically significant differences with p-values <0.05 were noted in univariate analysis for age (62.7 vs. 57.9 years, p=0.049) and need for renal replacement therapy (20.7% vs 5.3%, p=0.013) with regards to the outcome of mechanical ventilation. Neither age nor the need for renal replacement therapy showed significant findings with regards to ICU admission or in-hospital mortality. Comorbidities like diabetes, dyslipidemia, hypertension, heart disease and liver disease did not differ in prevalence when segregated into groups by any of the outcomes of interest.

In addition to the comorbidities and baseline characteristics, we recorded data on troponin I elevation and echocardiographic findings regarding left or right ventricular dysfunction. These factors were not independently associated with mortality, the need for mechanical ventilation, or ICU admission in univariate analysis. Troponin elevation did, however, achieve the threshold of p <0.10 to be included in the multivariate analysis for in-hospital mortality.

**Figure 1:**
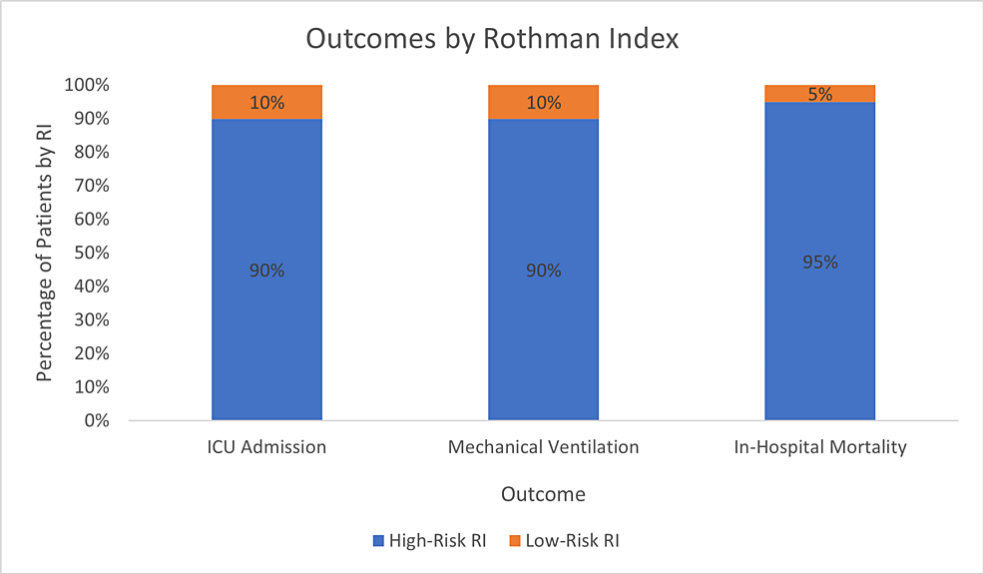
Outcomes by Rothman Index ICU, intensive care unit; RI, Rothman index.

Median RI score on admission for the entire cohort was 63.0 (IQR 45.0 - 77.1). Patients who died in the hospital had lower median RI scores than patients who survived (32.1 vs. 65.5, p <0.001). Lower RI scores were also associated with increased ICU utilization (45.9 vs. 70.0, p <0.001) and the need for mechanical ventilation (41.5 vs. 65.8, p <0.001). When RI scores were dichotomized into high-risk scores (RI <70) or low-risk scores (RI ≥70), p-values were <0.001 on univariate analysis for all three outcomes and hence, selected for multivariate analysis. 95.2% of patients who had a COVID-19-attributable death while in the hospital had a high-risk RI score. 90% of patients who were admitted to the ICU at some point during their hospital course had a high-risk RI score on admission. Similarly, 89.7% of patients that required mechanical ventilation had a high-risk RI score on admission (Figure 1).

Multivariate analyses are presented in Tables 2-4 for each of the selected outcomes. Obesity was independently associated with need for ICU admission (OR 2.16, 1.01 - 4.61; *p=*0.046). Age was not independently associated with any of the outcomes in multivariate analyses. Renal replacement therapy was not independently associated with the need for mechanical ventilation and troponin elevation was not independently associated with in-hospital mortality. A high-risk RI score (<70) was the only variable that showed consistent independent association with ICU utilization (OR 13.05, 4.66 - 36.57; p< 0.001), mechanical ventilation (OR 6.69, 1.88 - 23.78; p=0.003) and death (OR 12.58, 1.59 - 99.39; p=0.016).

**Table 2:** Multivariate Analysis of Predictors of Intensive Care Unit Admission in Patients Admitted with COVID-19 CI, confidence interval.

Variable	Odds Ratio	95% CI	*p*-value
High-Risk Rothman Index	13.05	4.66 - 36.57	0
Age	0.81	0.61 - 1.08	0.155
Obesity	2.16	1.01 - 4.61	0.046

**Table 3:** Multivariate Analysis of Predictors of Mechanical Ventilation in Patients Admitted with COVID-19 CI, confidence interval.

Variable	Odds Ratio	95% CI	*p-*value
High Risk Rothman Index	6.69	1.88 - 23.78	0.003
Age	0.89	0.65 - 1.24	0.497
Renal Replacement Therapy	3.04	0.94 - 9.88	0.065

**Table 4:** Multivariate Analysis for Predictors of In-Hospital Mortality in Patients Admitted with COVID-19 CI, confidence interval.

Variable	Odds Ratio	95% CI	*p-*value
High-Risk Rothman Index	12.58	1.59 - 99.39	0.016
Age	1.22	0.82 - 1.81	0.322
Elevated Troponin	1.44	0.52 - 3.94	0.481

## Discussion

When faced with the same disease, certain patients will have a more severe clinical course and many efforts have been made to find factors to separate individuals at increased risk of poor outcomes. Age is considered to be a major factor in disease mortality, but age may only be a proxy for the number and severity of comorbidities a given person may have. Scores like the Charlson comorbidity score have been created to account for the increased risk of any given patient [17]; similarly, comorbidities may in turn be a surrogate marker for acuity. A major outcome predictor for a given illness in patients with the same age and number of comorbidities may be their acuity, which can be evaluated by tools like the RI. RI holistically assesses a patient’s clinical status based on vital signs, laboratory values, and nursing assessments among other variables. Lower values correlate to more severe acuity. It has been used to detect early clinical deterioration to further improve care and identify patients at increased risk of mortality [6], but it has yet to be widely accepted in clinical practice. In this single-center retrospective study, we evaluated the association between RI on admission and relevant clinical outcomes to assess the potential utility of RI to predict important clinical outcomes in patients with a primary diagnosis of COVID-19. High-risk RI on admission was associated with higher mean hospital length of stay and led more frequently to ICU utilization, mechanical ventilation, and death.

As a novel disease, healthcare providers have not been able to reliably predict deterioration in patients with COVID-19. There are few predictive tools to stratify patients and guide care. Gong et al. conducted a retrospective review of 372 COVID-19 patients in China to identify patient characteristics and laboratory values on admission associated with developing the severe disease [18]. To improve risk stratification, they created a nomogram based on age, serum lactate dehydrogenase, C-reactive protein, coefficient of variation of red blood cell distribution width, blood urea nitrogen, direct bilirubin, and albumin levels. While this risk stratification tool may be used in patients with those laboratory values available on admission, it has limited use in patients without them, and it requires manual stratification of patients which is subject to human error. Additionally, given the differential expression of the angiotensin-converting enzyme 2 (ACE2) receptor protein for SARS-CoV-2 between populations [19], this prediction tool may not be valid for patient populations found in the United States. RI is an alternative method for evaluating clinical status that does not require all parameters to be filled and additionally, incorporates vital signs and nursing assessments.

The RI was created to assist clinicians in the early detection of patient deterioration [5]. It has been assessed as a predictive tool for outcomes in several clinical scenarios including both surgical and medical contexts. Alarhayem et al. performed a retrospective review of 1,445 surgical patients transferred from the surgical ICU (SICU) to the surgical floor and found that patients readmitted to the SICU had lower pretransfer and post-transfer RI scores [9], suggesting RI may assist in determining which patients are ready for discharge from the SICU to the surgical floor. In another study, among 2,687 patients who underwent elective spine surgery, RI was significantly inversely correlated with adverse events and readmission within 30 days of discharge [20]. Gotur et al. retrospectively evaluated 194 adult patients admitted to the medical ICU and found that patients with RI < 50 had a higher risk of adverse events post-discharge from the ICU [21]. Thus, RI may be useful in guiding medical ICU discharge decision-making. Additionally, in a prospective study, Arnold et al. compared physician judgment to RI for predicting clinical deterioration in a general hospital ward and found that the combination of physician judgment and RI outperformed either alone [11]. Another retrospective study of 227 patients with advanced cancer admitted to an institution showed RI scores <60 to be associated with longer length of stay, more palliative care, and hospice referrals, and increased mortality [22]. These studies highlight that RI may have predictive ability for several outcomes across different contexts, both surgical and medical. However, the efficacy of RI to predict patient deterioration in COVID-19 is not fully known.

Data is lacking in the evaluation of the association between RI scores and clinical outcomes in patients infected with SARS-CoV-2. Beals et al. conducted a multicenter retrospective study of 18,157 patients, 3,499 of which were infected with SARS-CoV-2 [23]. Admission RI provided the predictive ability of in-hospital mortality in both SARS-CoV-2-infected and non-SARS-CoV-2-infected patients. Stratification by admission of the RI into high-risk and low-risk groups also demonstrated increased mortality in high-risk patients. The discriminating ability of admission RI to predict the need for ICU admission was fair to good. The study highlights the predictive ability of admission RI to predict mortality and potentially ICU admission in patients admitted with COVID-19. Notably, the study does not differentiate patients admitted for COVID-19 management from patients admitted for other medical reasons who incidentally test positive for COVID-19 during admission. Thus, the predictive ability for mortality and ICU admission in this cohort may be confounded by patients experiencing other underlying medical conditions in the setting of asymptomatic SARS-CoV-2 infection. In the present study, patients admitted to our institution with a positive SARS-CoV-2 laboratory test were categorized based on the reason for admission. Patients who were not admitted for symptomatic COVID-19 were excluded from the study. Thus, the current study provides a better characterization of the predictive ability of admission RI for outcomes in the management of COVID-19.

While our study contributes to understanding the potential ability of admission RI to stratify patients and predict mortality, need for mechanical ventilation, and ICU admission, it has limitations warranting discussion. As a retrospective study, definitive conclusions may not be made about the predictive ability of RI but rather only about the associations between RI and outcomes. Confounding variables may not have been recorded during the retrospective review of the EMR, potentially influencing conclusions made from this study. Multivariate analysis helps to mitigate the role of confounding variables recorded but does not influence the effect of confounding variables that may have been missed.

## Conclusions

In conclusion, there are few applicable risk prediction models for the stratification of patients admitted for COVID-19. While healthcare provider judgment is essential to delivering high-quality care, a metric for early detection of patient deterioration in a novel disease such as COVID-19 can assist provider judgment. The RI provides a continuous variable assessing a patient’s clinical status and has been shown in several non-COVID-19 contexts to have stratification abilities for outcomes such as ICU readmission, hospital readmission, and mortality. Data evaluating the utility of RI in patients admitted for COVID-19 is lacking. In this study, stratification of admission RI by severity showed that high-risk admission RI in COVID-19 was independently associated with mortality, the need for mechanical ventilation, and ICU admission, and had more predictive value than traditional risk factors. Low admission RI scores should prompt healthcare providers to have heightened vigilance and a lower threshold for advancing care unless provider judgment determines further care to be futile. Prospective studies may be helpful to evaluate the incorporation of RI into clinical practice and its effects on clinical decision-making, resource utilization, and clinical outcomes. Furthermore, the association between admission RI and outcomes associated with new coronavirus variants as well as RI’s predictive ability for vaccinated and unvaccinated individuals should be explored.
